# Bacterial Membrane-Derived Vesicles Attenuate Vancomycin Activity against Methicillin-Resistant *Staphylococcus aureus*

**DOI:** 10.3390/microorganisms9102055

**Published:** 2021-09-29

**Authors:** Monika Kumaraswamy, Kamilla Wiull, Bishnu Joshi, George Sakoulas, Armin Kousha, Gustav Vaaje-Kolstad, Mona Johannessen, Kristin Hegstad, Victor Nizet, Fatemeh Askarian

**Affiliations:** 1 Infectious Diseases Section, VA San Diego Healthcare System, San Diego, CA 92161, USA; 2 Department of Medicine, University of California San Diego, La Jolla, CA 92093, USA; 3 Collaborative to Halt Antibiotic Resistant Microbes (CHARM), University of California San Diego, La Jolla, CA 92093, USA; vnizet@health.ucsd.edu; 4 Faculty of Chemistry, Biotechnology and Food Science, Norwegian University of Life Sciences, 1433 Ås, Norway; kamilla.wiull@nmbu.no (K.W.); gustav.vaaje-kolstad@nmbu.no (G.V.-K.); 5 Research Group for Host-Microbe Interactions, UiT-The Arctic University of Norway, 9037 Tromsø, Norway; joshi.bishnu@uit.no (B.J.); mona.johannessen@uit.no (M.J.); kristin.hegstad@uit.no (K.H.); 6 Department of Pediatrics, University of California San Diego, La Jolla, CA 92093, USA; gsakoulas@health.ucsd.edu (G.S.); armin.kousha@gmail.com (A.K.); 7 Norwegian National Advisory Unit on Detection of Antimicrobial Resistance, University Hospital of North-Norway, 9038 Tromsø, Norway; 8 Skaggs School of Pharmacy and Pharmaceutical Sciences, University of California San Diego, La Jolla, CA 92093, USA

**Keywords:** methicillin-resistant *Staphylococcus aureus*, vancomycin, membrane vesicles

## Abstract

Methicillin-resistant *Staphylococcus aureus* (MRSA) has evolved numerous antimicrobial resistance mechanisms and is identified as a serious public health threat by the World Health Organization and U.S. Centers for Disease Control and Prevention. The glycopeptide vancomycin (VAN) remains a cornerstone of therapy for severe MRSA infections despite increasing reports of therapeutic failure in hospitalized patients with bacteremia or pneumonia. Recently, the role of released bacterial-derived membrane vesicles (MVs) in antibiotic resistance has garnered attention. Here we examined the effect of exogenous MRSA-derived MVs on VAN activity against MRSA in vitro, using minimum inhibitory concentration and checkerboard assays, and ex vivo, incorporating components of host innate immunity such as neutrophils and serum complement present in blood. Additionally, the proteome of MVs from VAN-exposed MRSA was characterized to determine if protein expression was altered. The presence of MVs increased the VAN MIC against MRSA to values where clinical failure is commonly observed. Furthermore, the presence of MVs increased survival of MRSA pre-treated with sub-MIC concentrations of VAN in whole blood and upon exposure to human neutrophils but not human serum. Unbiased proteomic analysis also showed an elevated expression of MV proteins associated with antibiotic resistance (e.g., *marR*) or proteins that are functionally linked to cell membrane/wall metabolism. Together, our findings indicate MRSA-derived MVs are capable of lowering susceptibility of the pathogen to VAN, whole-blood- and neutrophil-mediated killing, a new pharmacodynamic consideration for a drug increasingly linked to clinical treatment failures.

## 1. Introduction

*Staphylococcus aureus* (SA) are Gram-positive cocci that colonize healthy and immunocompromised individuals in both community and hospital settings. In one European study evaluating the anterior nares of 3464 patients, nearly 50% were identified to be SA carriers [1]. A major cause of human infection-related morbidity and mortality, SA is associated with skin and soft tissue infections and invasive diseases including bacteremia, infective endocarditis, necrotizing pneumonia, intravascular line infection, prosthetic joint infection, osteomyelitis and toxic shock syndrome [2]. SA pathogenesis is driven by numerous virulence factors including secreted toxins (e.g., α, β, γ, δ, exfoliative, enterotoxin), phospholipase C, metalloproteases, capsular polysaccharide, protein A, fatty acid modifying enzyme and lipases, V8 protease, leukocidins, phenol soluble modulins, its golden pigment and staphylokinase [3,4].

Recently, bacterial-derived membrane vesicles (MVs) have been implicated in the pathogenesis of SA infections [5]. The spherical bi-layered MVs originate from the blebbing of bacterial outer membranes in Gram-negative bacteria or by phage endolysin-triggered cell lysis in Gram-positive bacteria [6,7]. MVs can harbor diverse cargo including biologically active toxins, β-lactamases, polysaccharides, nucleic acids and other cellular metabolites [8,9]. MVs can stimulate biofilm formation, act as immunomodulators, increase bacterial resistance to host whole-blood killing, neutrophil killing, serum-complement killing, endogenous antimicrobial peptides and pharmaceutical antibiotics, and may in part be mediated by phenol-soluble modulins [10,11]. Previous studies suggest MVs confer certain SA protection against the lipopeptide membrane-active antibiotic daptomycin in vitro and ex vivo in whole blood, and that certain antimicrobial agents (e.g., gentamicin, cefotaxime, ampicillin and imipenem) can trigger bacterial MV release, potentially yielding antimicrobial resistance [12,13,14]. However, the role of methicillin-resistant *Staphylococcus aureus-* (MRSA) derived MVs and its implications in resistance to the first line therapeutic glycopeptide antibiotic vancomycin (VAN) has yet to be explored.

MRSA appeared shortly following the introduction of methicillin in 1960 [15]. VAN was developed soon thereafter and emerged as the primary therapy for serious MRSA infections, with most isolates continuing to exhibit susceptible minimum inhibitory concentration (MIC) testing criteria as defined by the Clinical and Laboratory Standards Institute (CLSI) and the European Committee on Antimicrobial Susceptibility Testing (EUCAST), ranging between 0.5 and 2.0 mg/L. VAN inhibits bacterial cell wall synthesis by binding to the d-Ala-d-Ala C peptidoglycan terminus on the outer surface of the cytoplasmic membrane, preventing further elongation and cross-linking of the peptidoglycan matrix [16]. However, despite almost uniform microbiological susceptibility, clinical treatment failures are increasingly common, particularly in high-inoculum respiratory and endovascular infections [16,17]. Our study explored whether exogenous MRSA-derived MVs protect the bacterium against VAN killing, or influence its clearance by neutrophils or serum complement. We also studied whether the selection pressure of sub-therapeutic concentrations of VAN altered the expression of MV proteinaceous content in MRSA using proteomics-based characterization.

## 2. Materials & Methods

### 2.1. Bacterial Strains, Media and Antibiotics

MRSA USA 300 strain ATCC BAA-1717 (i.e., TCH1516) was used in all experiments and stored in Brain Heart Infusion (BHI) broth (Hardy Diagnostics, Santa Maria, CA, USA) with 50% glycerol at −80 °C until the study. Mueller–Hinton broth (Spectrum Chemicals, New Brunswick, NJ, USA) supplemented with 25–50 mg/L Ca^2+^ (Sigma-Aldrich, Taufkirchen, Germany) and 10–12.5 mg/L Mg^2+^ (Sigma-Aldrich, Taufkirchen, Germany) (CA-MHB) served as the standard bacteriologic growth and MIC testing medium, while additional studies were performed in mammalian tissue culture medium Roswell Park Memorial Institute 1640 (ThermoFisher Scientific, MA, USA) with added 5% BHI broth (RPMI + 5%BHI). The antibiotics vancomycin (VAN), daptomycin (DAP), cefazolin (CFZ) and nafcillin (NAF) were purchased from Sigma-Aldrich (Taufkirchen, Germany). Note: Assays performed with DAP included a Ca^2+^ concentration of 50 mg/L to ensure appropriate antibiotic activity.

### 2.2. Isolation and Purification of MVs

MRSA grown overnight at 37 °C in BHI broth were diluted 1:100 (5/500 mL) in fresh BHI supplemented +/− sub-therapeutic VAN (0.5 mg/L). Freshly inoculated cultures +/− VAN were grown to stationary phase (incubated at 37 °C for 16–17 h) for MV isolation, and performed as previously described with the following modifications [5]: The culture supernatant was centrifuged at 6000× *g* for 30–40 min at 4 °C and filtered through a 0.22 μm vacuum filter (Merck Millipore, Darmstadt, Germany) to remove residual cellular debris. The supernatant was then ultra-centrifuged at 100,000× *g* at 4 °C for at least 3 h using either 45 or 50.2 Ti rotors (Beckman Coulter, Brea, CA, USA). The MV pellets obtained following several rounds of isolation were then pooled, washed twice with phosphate-buffered saline (Gibco PBS, Life Technologies, Carlsbad, MA, USA) following ultra-centrifugation at 100,000× *g* for 3 h at 4 °C, and resuspended in fresh PBS. To ensure sterility, an aliquot of MVs was spread on BHI agar and incubated overnight at 37 °C to verify the absence of microbial growth. Isolated MVs were then stored at −80 °C until usage. MVs isolated in the absence of VAN were utilized in all experiments performed (checkerboards, serum killing, neutrophil killing, and whole blood killing). Only proteomic analysis was conducted using both MVs isolated in the absence and presence of sub-inhibitory VAN to help determine if antibiotic exposure altered protein expression (e.g., virulence factors and mechanisms of antimicrobial resistance).

For the proteomic analysis, pooled MVs underwent an extra fractionation step with density gradient centrifugation using OptiPrep (Sigma-Aldrich, Taufkirchen, Germany) as previously described with minor modifications [5]. Varying amounts of OptiPrep (in PBS) were sequentially added to an ultracentrifugation tube (final volume: 5 mL) from bottom to top as follows: 45% (400 μL), 35% (600 μL), 30% (600 μL), 25% (600 μL), 20% (600 μL), 15% (500 μL), and 10% (400 μL). Lastly, isolated MVs were then added to the top of the tube and subjected to ultra-centrifugation at 100,000× *g* for 3h at 4 °C in slow acceleration and deceleration mode to obtain a stable layer of MV ring formation. Thereafter, 200 μL aliquots (total of 25 fractions) were sequentially collected and subjected to SDS-PAGE (Life Technologies, Waltham, MA, USA) followed by Coomassie blue staining. Fractions showing the same protein profile on Coomassie stained gel were pooled (Figure S1), and submitted for transmission electron microscopy (Figure S2 and Supplementary Materials and Methods) and proteomics analysis. Concentrations of MVs containing protein were quantified using a Pierce BCA Protein Assay Kit (ThermoFisher Scientific, MA, USA) or Direct Detector^TM^ (Merck Millipore, Germany). Additionally, an Invitrogen Qubit Protein Assay Kit (MA, USA) was used for the preliminary estimation of protein concentration in the proteomic analysis per the manufacturer’s instructions.

### 2.3. Minimum Inhibitory Concentration and Checkerboard Assays

Broth microdilution MIC and checkerboard assays were performed in CA-MHB in accordance with CLSI guidelines, and RPMI + 5%BHI using antibiotics (VAN, DAP, CZN and NAF) or MVs alone or in combination [18]. The concentrations of antibiotics evaluated via MIC assays ranged from 0.5–64 mg/L. The concentrations of antibiotics and MVs evaluated in combination via checkerboard assays ranged from 0–8 mg/L or 0–64 mg/L and 0–100 mg/L, respectively.

### 2.4. Ethical Approval

Human whole blood (and the isolation of associated components including neutrophils and serum) was obtained from consenting healthy donors under a protocol (131002X) approved by the UC San Diego Human Subjects Institutional Review Board.

### 2.5. Whole Blood Killing Assay

MRSA viability in human whole blood was assessed as previously described with the following modifications [19]: Freshly drawn human blood anticoagulated with heparin (160 µL) was mixed with 2 × 10^4^ CFU MRSA (20 µL) grown overnight in the absence or presence of sub-inhibitory VAN (0.5 mg/L), or grown overnight and subsequently co-incubated with VAN (0.5 mg/L), 20 μL of PBS and/or 20 μL of MVs (final concentration of 10 μg per 100 µL) in siliconized tubes (Sigma-Aldrich, Taufkirchen, Germany). Tubes were incubated for 3 h at 37 °C on a rotator. Next, serial dilutions of samples were performed using sterile ice-cold MQ water + Saponin (0.3%) (Sigma Aldrich, Taufkirchen, Germany), and were plated on Todd Hewitt Agar (THA) plates for bacterial enumeration and calculation of percentage survival vs. the initial inoculum.

### 2.6. Neutrophil Killing Assay

Neutrophil bactericidal assays were performed as previously described with the following modifications [18]: Human neutrophils were isolated from healthy donors using the PolymorphPrep system (Axis-Shield, Oslo, Norway). Freshly isolated neutrophils were resuspended to 2 × 10^6^ cells/mL in RPMI and used to seed a 96-well plate (2 × 10^5^ cells/well). MRSA and/or purified MVs (final concentration of 10 μg per 100 µL) were added to the applicable wells. Neutrophils were infected at a multiplicity of infection (MOI) = 1 using MRSA grown overnight in the absence or presence of a sub-inhibitory concentration of VAN (0.5 mg/L). Plates were centrifuged at 500× *g* for 10 min prior to a 15- or 30-min incubation at 37 °C with 5% CO_2_. Serial dilutions of samples were performed using sterile PBS + Triton-X 100 (0.02%) for bacterial enumeration and calculation of percentage survival vs. the initial inoculum.

### 2.7. Serum Killing Assay

Serum killing assays were performed using pooled serum obtained from three healthy donors and as previously described with the following modifications [20]: MRSA grown overnight with or without 0.5 mg/L VAN (1.8 × 10^5^ CFU/well) were added to RPMI ±10% pooled human serum. Thereafter, PBS or MVs (final concentration of 10 μg per 100 µL) were added to samples. Assays were performed with a final volume of 300 µL using siliconized tubes rotated in a 37 °C incubator for 1h before completing serial dilutions with sterile PBS followed by plating on THA for CFU enumeration. The survival index was defined as CFU enumerated at the end of the assay divided by CFU present at the 0 h time point.

### 2.8. Growth Curve

MRSA inoculated in 5 mL of BHI or CA-MHB were grown overnight to stationary phase (14–16 h) at 37 °C in a shaking incubator. The next day, the bacteria were washed twice with PBS and resuspended in fresh BHI or CA-MHB to 1 × 10^6^ CFU/mL +/− VAN (0.5 mg/L) and used to seed a 96-well flat bottom plate (1 × 10^5^ CFU/well). Plates were subsequently placed in a Bioscreen C MBR reader (Oy Growth Curves Ab Ltd., Turku, Finland) maintained at 37 °C with re-growth assessed by OD_600_ every 6 h.

### 2.9. Proteomic and Bioinformatic Analysis of MVs Containing Protein

In-solution digestion and liquid chromatography mass spectrometry (MS) were performed by UiT-The Arctic University of Norway’s Proteomic Core Facility using MVs isolated from MRSA in the absence and presence of VAN as described earlier [21,22]. The protein pellet (20 µg) was resuspended in 8 M urea, reduced with 20 mM dithiothreitol (DTT), and alkylated by treatment with 40 mM iodoacetamide prior to trypsinization (1:20) (Promega, Madison, WI, USA). Peptide purification and desalting was performed using OMIX C18 tips (Varian, Crawley, CA, USA). The purified peptide was dissolved in 0.1% formic acid and injected on an Easy-nLC autosampler (Thermo Fisher Scientific, Waltham, MA, USA) containing an EASY-Spray column (C18, 2 μm, 100 Å, 50 μm, 50 cm). Nano liquid chromatography (LC) was run at a flow rate of 250 nL/min containing 2–100% acetonitrile gradient in 0.1% formic acid over 50 min. Separated peptides were analyzed using a Q-Exactive mass spectrometer (Thermo Fisher Scientific, Waltham, MA, USA). Data were collected in data-dependent mode using a Top10 method. Raw data were searched against SA *M1516* (from NCBI) using the Sequest algorithm in Proteome Discoverer version 2.1 [23]. Peptide mass tolerances used in the search were 10 ppm, and fragment mass tolerance was 0.02 Da. Peptide ions were filtered using a false discovery rate (FDR) set to 5% for protein identifications. Networking and enrichment analyses were performed using STRING database [24]. For the appropriate input, GI numbers were converted to the corresponding UniProtKB ID; in total, 521 of 530 MV-associated proteins were mapped into UniProtKB ID. Virulence factors were selected either from the list provided in the following link: http://www.mgc.ac.cn/cgi-bin/VFs/compvfs.cgi?Genus=Staphylococcus (23 September 2021) or through a literature review.

### 2.10. Statistical Analysis

Data were represented as the mean ± SEM of three experiments unless otherwise specified. Two-way analyses of variance were used where appropriate. *p*-values < 0.05 were regarded to be statistically significant. All statistical analyses were performed using GraphPad Prism 8.0 (GraphPad Software).

## 3. Results

### 3.1. Exogenous MVs Promote MRSA Survival in the Presence of VAN

**Figure 1:**
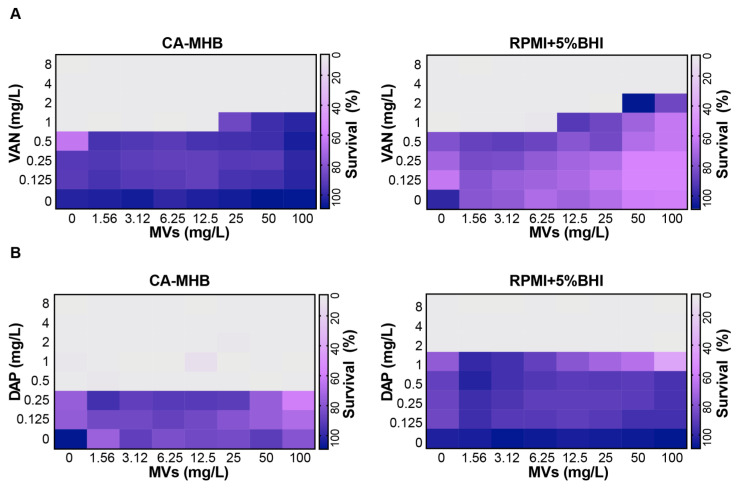
MVs potentiate MRSA survival in the presence of VAN. Antimicrobial susceptibility of MRSA to (**A**) VAN or (**B**) DAP in combination with MVs was assessed by checkerboard broth microdilution assays performed in CA-MHB or RPMI + 5%BHI. Bacterial growth quantified by OD_600_ and compared to the growth of untreated control wells was reflected as a heat map of percentage bacterial survival. Data are representative of at least two biologic replicates. CA-MHB: Cation-adjusted Mueller–Hinton Broth; RPMI + 5%BHI: Roswell Park Memorial Institute 1640 + 5% Brain Heart Infusion; VAN: Vancomycin; DAP: Daptomycin; MVs: Membrane Vesicles.

MIC and checkerboard assays were performed using cell-membrane/wall-targeting antibiotics DAP, NAF, CFZ and VAN alone or in combination with exogenous MRSA-derived MVs and the clinical strain MRSA TCH1516. Studies were performed in the bacteriologic media CA-MHB, or in the supplemented mammalian tissue culture media RPMI + 5%BHI (Table 1). The DAP (2 vs. 0.5 mg/L), NAF (64 vs. 4 mg/L), and CFZ (64 vs. 4 mg/L) MICs were notably lower using RPMI + 5%BHI compared to CA-MHB but unchanged for VAN (1 vs. 1 mg/L). Exposure to exogenous MRSA-derived MVs (25–100 or 50–100 mg/L) together with VAN, an antibiotic commonly deployed in the management of SA infections, increased the MIC of VAN from a susceptible MIC of 1 mg/L to a more resistant MIC of 2 (borderline susceptible) and 4 mg/L (resistant) in CA-MHB and RPMI + 5%BHI, respectively, and based on clinical breakpoints determined by CLSI and EUCAST (Figure 1A). In contrast, the concomitant presence of MVs did not increase the MICs of NAF, CFZ or DAP in either medium (Figure 1B and Table 1).

### 3.2. MVs, VAN and Innate Immune Mediated Killing of MRSA

**Figure 2:**
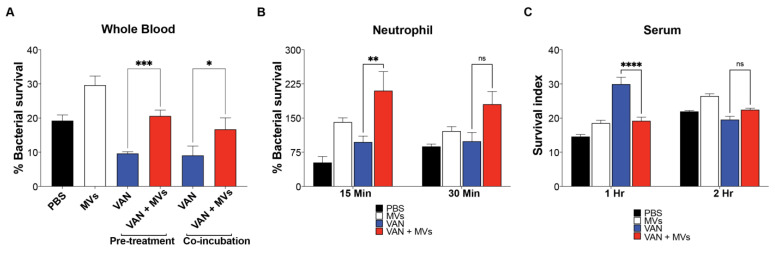
Ex vivo analysis of MRSA, VAN, MVs and components of host innate immunity. MRSA grown overnight or co-incubated with 0.5 mg/L of VAN are more resistant to killing by (**A**) human whole blood (3 h) and (**B**) isolated neutrophils (15 and 30 min) in the presence of MVs (20 µg). However, exogenous MVs did not significantly increase the killing of MRSA pre-treated overnight with 0.5 mg/L of VAN to (**C**) human serum complement (1 and 2 h). Data are representative of the mean ± SEM from a combination of three experiments performed in triplicate. * *p* < 0.05, ** *p* < 0.01, *** *p* < 0.001, **** *p* < 0.0001, or no statistical significance (ns) in VAN treated samples by two-way ANOVA. PBS: Phosphate-Buffered Saline; MVs: Membrane Vesicles; VAN: Vancomycin.

MV enhancement of MRSA survival in the presence of VAN was further evaluated in the context of host innate defenses by ex vivo assays incorporating whole blood, neutrophils or serum complement. Direct co-incubation of MRSA with 0.5 mg/L VAN and 20 µg of MVs increased bacterial survival in freshly isolated human whole blood that harbors multiple components of innate immunity including leukocytes, platelets and serum complement (Figure 2A). Furthermore, the presence of MVs also promoted resistance of MRSA pre-treated overnight with sub-MIC concentrations of VAN (0.5 mg/L) to killing by whole blood and purified human neutrophils but not by serum complement (Figure 2A–C). Our ex vivo studies suggest increased MRSA survival in the presence of MV may be partially mediated by encumbrances to neutrophil killing.

### 3.3. VAN Exposure Influences MRSA MV Proteome Content

**Figure 3:**
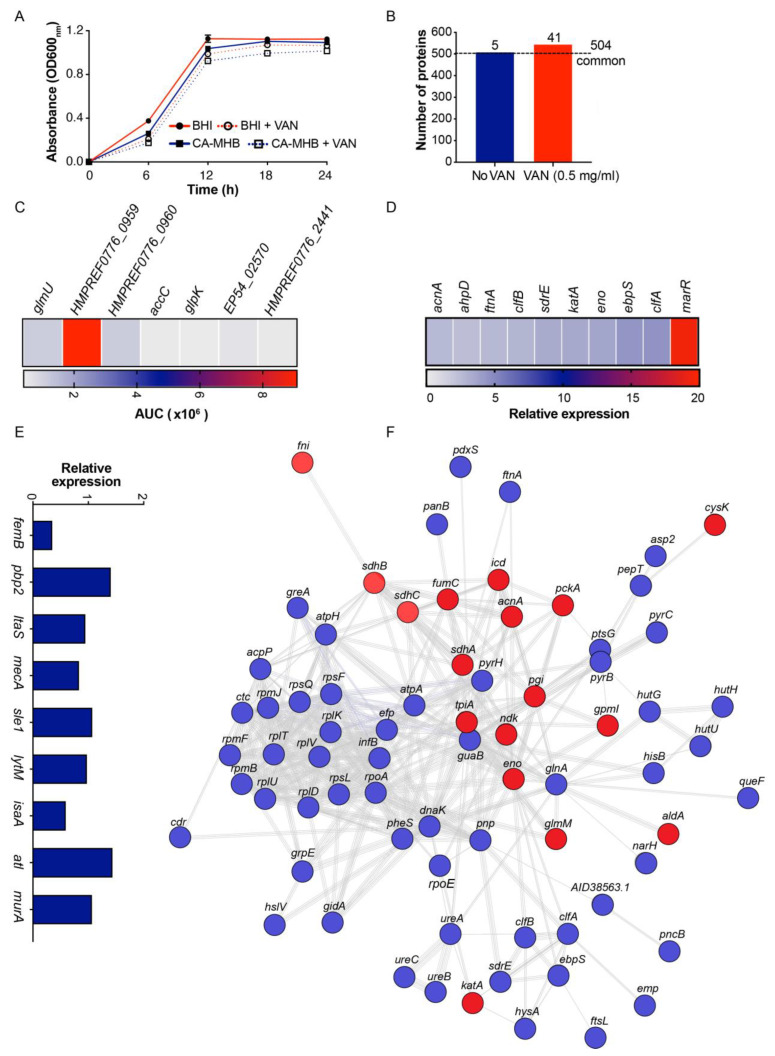
Proteomic profile of MV peptides derived from VAN pre-treated MRSA. (**A**) Growth of MRSA in BHI or CA-MHB +/− VAN (0.5 mg/L) at 24 h was not impeded by the presence of sub-MIC VAN. (**B**) Number of proteins identified from MVs isolated from untreated or VAN pre-treated MRSA. Heat maps illustrating unique MV proteins associated with (**C**) cell wall or cell membrane metabolism and (**D**) virulence from MRSA pre-treated with VAN are represented as the area under the curve (AUC) or relative expression (>2-fold expression relative to proteins identified from untreated MRSA-derived MVs), respectively. (**E**) Expression of proteins involved in cell wall biosynthesis (e.g., peptidoglycan and lipoteichoic acid) from MV proteins derived from VAN pre-treated MRSA relative to untreated MRSA. (**F**) Functional STRING and KEGG analyses of proteins expressed under selective pressure from sub-MIC VAN demonstrated a highly interconnected network of proteins associated with general metabolic processes (gray), virulence (blue) and antibiotic resistance (red). Singletons or chains with no interaction within the main network have been deleted. Gene nomenclatures are depicted in Supplementary Dataset 1 and 2. MVs: Membrane Vesicles; BHI: Brain Heart Infusion; CA-MHB: Cation-adjusted Mueller–Hinton Broth; VAN: Vancomycin; HMPREF0776_0959: Acetyl-CoA C-acetyltransferase; HMPREF0776_0960: 3-hydroxyacyl-CoA Dehydrogenase; EP54_02570: Lipoprotein; HMPREF0776_244: Peptidase (S41 Family).

The MS/MS spectra of MV proteins isolated from MRSA TCH1516 untreated or pre-treated overnight with a sub-bacteriostatic concentration of (0.5 mg/L) VAN were compared vs. the well-annotated genome of SA *M1516*. Pre-treatment with VAN did not impede the growth of MRSA in BHI or CA-MHB (Figure 3A). A total of 509 and 545 proteins were identified from MVs isolated from untreated or VAN pre-treated MRSA grown in BHI (Figure 3B and Supplementary Dataset Supplementary 1), respectively. Ultimately, 504 proteins were assigned to a common proteome, and notably, 41 unique proteins were associated with VAN exposure (Table 2). Some of the identified unique proteins are associated with VAN pre-treatment including N-acetylglucosamine-1-phosphate uridyltransferase (*glmU*), acetyl-CoA biotin carboxylase (*accC*), glycerol kinase (*glpK*), and proteases (*htrA* and SAUSA300_0816) previously found to be upregulated in response to other cell-wall-damaging antibiotics (oxacillin, cycloserine, bacitracin, etc.) and are functionally linked to cell membrane/wall metabolism or maintenance of important periplasmic and membrane proteins (Figure 3C and Supplementary Dataset 1) [25,26].

The abundance of several virulence factors with established roles in SA pathogenesis (e.g., clumping factor A (*clfA*), clumping factor B (*clfB*), serine aspartate repeat containing protein E (*sdrE*), elastin binding protein (*ebpS*)), evasion of host oxidative stress (e.g., alkyl hydroperoxide reductase (*ahpD*), bacterial non-heme ferritin (*ftnA*), catalase (*katA*)) and bacterial detoxification to diverse antibiotics including norfloxacin and ampicillin (by the multiple antibiotic resistance regulator (*marR*) family of transcription factors) were markedly increased (>2- to 19-fold change) in the MVs isolated from MRSA pre-treated with VAN relative to untreated MRSA (Figure 3D and Supplementary Dataset 2). Furthermore, penicillin binding protein 2 (PBP2), whose expression is essential for VAN glycopeptide resistance and cell wall biosynthesis, was also increased by 1.4-fold in MVs derived from MRSA exposed to VAN (Figure 3E) [27,28,29]. A KEGG network analysis of proteins highly expressed in MVs from VAN pre-treated MRSA were highly interconnected and predominantly associated with metabolic processes, virulence, and antibiotic resistance (Figure 3F). Our data indicate that the elevated relative expression of proteins captured in the released membrane vesicles of VAN pre-treated MRSA reflects a bacterial proteomic response that is modulating antibiotic and innate immune susceptibility.

## 4. Discussion

VAN remains a first-line antibiotic in the treatment of severe MRSA infection based on high rates of susceptibility identified by MIC testing and its low cost [30]. However, increased mortality and treatment failures have been seen in patients with MRSA bacteremia and hospital-acquired MRSA infections treated with VAN irrespective of the corresponding MIC [17,31,32]. Our study suggests MV and MV-associated proteins produced in response to antibiotic exposure and environmental stressors may influence VAN susceptibility by functioning as a cell wall/membrane decoy to sequester the antibiotic, and may also have ramifications on the pathogen’s susceptibility to neutrophil and whole-blood killing.

MIC and checkerboard testing revealed the presence of MVs in standard bacteriologic (CA-MHB) and supplemented mammalian tissue culture (RPMI + 5%BHI) mediums raised the MIC of VAN from 1 mg/L to 2 mg/L and 2 mg/L to 4 mg/L, respectively (Figure 1A). The current clinical MIC breakpoint of VAN for MRSA is 2 mg/L according to EUCAST and CLSI guidelines, so the magnitude of observed MIC elevation approached or exceeded the clinical breakpoint where clinical failure is often observed and supplemental or alternative antibiotic regimens are typically pursued [31,33,34].

Conversely, the presence of MVs did not influence the MIC of DAP, an alternative agent for MRSA infection, in either CA-MHB or RPMI + 5%BHI (Figure 1B). Loss of the accessory gene regulator (*agr*) quorum sensing system, a regulator of virulence gene expression, enhances MRSA resistance to DAP by shedding membrane phospholipids [13]. However, the virulent MRSA TCH1516 used in our investigation harbored an intact *agr* locus [35]. Although MRSA harboring *agr* release membrane phospholipids in response to DAP, they also secrete phenol-soluble modulins, small cytolytic toxins that counteract DAP inactivation. Pader et al. demonstrated that SA phospholipid shedding was inhibited by oxacillin (OXA), a β-lactam antibiotic used in combination with DAP as salvage therapy in recalcitrant MDR staphylococcal infections, boosting bacterial killing in experimental in vivo models [13].

The relevance of our findings with VAN were extended to interactions with innate immunity. Ex vivo assays using human whole blood revealed MVs enhanced survival of MRSA co-incubated or pre-treated overnight with VAN compared to that of MRSA in the absence of antibiotics (Figure 2A). Additionally, the presence of MVs promoted resistance of MRSA pre-treated with VAN to neutrophil, but not serum killing (Figure 2B,C). Proteomic analysis of MVs identified catalase (*katA*), alkyl hydroperoxide reductase (*ahpC*, *ahpF*), leukocidins (*lukE*, *lukS*) and *α*-hemolysin (*hla*), enzymes known to reduce oxidative burst triggered by phagocytic cells such as neutrophils, and potent toxins known to target and lyse host immune cells [36].

Furthermore, MV proteomic analysis of VAN-treated MRSA showed increased expression of virulence factors predominantly associated with adhesion and colonization of the nares, skin, elastin-rich tissues and foreign bodies, bacterial clumping and fibrin clot formation including clumping factors A and B, serine aspartate repeat containing protein E, and elastin-binding protein. Indeed, the interesting epidemiological association of MRSA colonization being associated with prior glycopeptide exposure may be attributed at least in part by these observations [37]. Higher levels (>19-fold) of the *marR* family of transcription factors, proteins known to facilitate resistance to β-lactams, quinolones or whose upregulation have been implicated in VAN resistance, were also detected [27,28,29,38].

In conclusion, these findings highlighting MV shedding by SA may represent an adaptive mechanism against VAN exposure and may increase the concentration of VAN required to achieve bacterial clearance. Our investigation represents a first step in exposing the potential involvement of MVs in VAN therapeutic failure observed in severe MRSA infections, and may be another pharmacodynamic consideration in the evaluation of VAN vs. alternative mono and dual therapies (such as DAP, or DAP + OXA) which are potentially less susceptible to being thwarted by MV release. Future studies including multiple clinical MRSA strains obtained from infections involving VAN therapeutic failure, quantification of MV production in high-burden infections, visualization of antibiotic entrapment by MVs, and in vivo studies must be performed to address the limitations of our preliminary proof-of-principle studies and assess the broader applicability of our findings.

## Figures and Tables

**Table 1:** Antimicrobial susceptibility of methicillin-resistant *Staphylococcus aureus* (MRSA) to cell-wall- or cell-membrane-targeting antibiotics in the absence and presence of exogenous bacterial-derived membrane vesicles (MVs) determined using minimum inhibitory concentration (MIC) and checkerboard assays.

MRSA (TCH 1516)	−MVs (0 mg/L)				+MVs (1.56–100 mg/L)			
	VAN	DAP	CFZ	NAF	VAN ^a^	DAP	CFZ	NAF
CA-MHB	1	2	≥64	≥64	2	2	≥64	≥64
RPMI + 5%BHI	1	0.5	4	4	4	0.5	4	4

Data are represented as mg/L. ^a^ VAN MIC increased from 1 to 2 mg/L and 1 to 4 mg/L in the presence of MV concentrations ranging from 25–100 mg/L and 50–100 mg/L in CA-MHB and RPMI + 5%BHI, respectively. MRSA: Methicillin-Resistant *Staphylococcus aureus*; MVs: Membrane Vesicles; MIC: Minimum Inhibitory Concentration; CA-MHB: Cation-adjusted Mueller–Hinton Broth; RPMI + 5%BHI: Roswell Park Memorial Institute 1640 + 5% Brain Heart Infusion; VAN: Vancomycin; DAP: Daptomycin; CFZ: Cefazolin; NAF: Nafcillin.

**Table 2:** Unique proteins associated with MVs isolated from MRSA, grown in the presence of sub-MIC VAN (0.5 mg/L).

Gene Symbol/ORF	Description
*dut*	dUTP diphosphatase
*purE*	N5-carboxyaminoimidazole ribonucleotide mutase
*valS*	Valine—tRNA ligase
*rocF*	Arginase
*SAUSA300_2144*	Uncharacterized protein
*glmU*	Bifunctional protein GlmU
*HMPREF0776_2553*	Dehydrogenase E1 component
*alaS*	Alanine—tRNA ligase
*HMPREF0776_0959*	Acetyl-CoA C-acetyltransferase
*SAUSA300_2328*	Uncharacterized protein
*dmpI*	Tautomerase
*mtlD*	Mannitol-1-phosphate 5-dehydrogenase
*SAUSA300_0003*	Uncharacterized protein
*SAUSA300_1690*	Putative thioredoxin
*HMPREF0776_2533*	Alpha-amylase
*SAUSA300_0289*	Uncharacterized protein
*lysS*	Lysine—tRNA ligase
*pyrG*	CTP synthase
*SAUSA300_0706*	Putative osmoprotectant ABC transporter, ATP-binding protein
*SA0224*	3-hydroxyacyl-CoA dehydrogenase, NAD binding domain protein
*recA*	Protein RecA
*HMPREF0776_0345*	Accessory regulator family
*SAUSA300_2132*	UPF0457 protein SAUSA300_2132
*accC*	Acetyl-CoA carboxylase, biotin carboxylase
*HMPREF0776_2830*	Excalibur domain protein
*SAUSA300_0460*	Uncharacterized protein
*hflB ftsH*	ATP-dependent zinc metalloprotease FtsH
*SAUSA300_2289*	Uncharacterized protein
*isdB*	Iron-regulated surface determinant protein B
*srrA*	Staphylococcal respiratory response protein, SrrA
*codY*	GTP-sensing transcriptional pleiotropic repressor CodY
*SAUSA300_0816*	UPF0337 protein SAUSA300_0816
*rpsD*	30S ribosomal protein S4
*HMPREF0776_2441*	Peptidase, S41 family
*rpsR*	30S ribosomal protein S18
*HMPREF0776_1767*	HD domain protein
*SAUSA300_1674*	Putative serine protease HtrA
*HMPREF0776_0647*	Uncharacterized protein
*EP54_02570*	Lipoprotein
*glpK*	Glycerol kinase
*HMPREF0776_2430*	Glyoxalase family protein

MRSA: Methicillin-Resistant *Staphylococcus aureus*; MIC: Minimum Inhibitory Concentration; MVs: Membrane Vesicles.

## Data Availability

Proteomic data have been uploaded to the Proteomics Identification Database (PRIDE) (PXD024232).

## Supplementary Materials

The following are available online at https://www.mdpi.com/article/10.3390/microorganisms9102055/s1. Supplementary Materials for this article include Supplementary Materials and Methods (Transmission Electron Microscopy Analysis), Supplementary Figures S1–S2, Dataset legend, Supplementary References, and Supplementary Datasets (1–2). Figure S1: Coomassie blue stained protein gel of pooled MRSA-derived MVs fractions. Figure S2: TEM of MVs isolated from MRSA (TCH1516) after OptiPrep. Dataset 1: List of identified MRSA-derived MV proteins obtained from bacteria grown in BHI in the absence or presence of sub-MIC VAN (0.5 mg/L). The results were obtained from pooled MVs purified from multiple isolations. Protein abundance is represented as the area under the curve (AUC). Dataset 2: List of identified MRSA-derived MV proteins with increased expression (>2 fold) in the presence of sub-MIC VAN relative to no VAN.
