# Supplementary material for: Bacterial Membrane-Derived Vesicles Attenuate Vancomycin Activity against Methicillin-Resistant Staphylococcus aureus
Source: Microorganisms. 2021 Sep 29;9(10):2055. doi: 10.3390/microorganisms9102055 (PMC8539228; doi:10.3390/microorganisms9102055)
Supplement: Supplementary file 1 [file microorganisms-09-02055-s001.zip › SUPPLEMENTARY_MATERIAL.pdf]

SUPPLEMENTARY MATERIAL

SUPPLEMENTARY MATERIALS AND METHODS

Transmission electron microscopy (TEM) analysis. Five µL of purified MVs were subjected to negative stating for TEM analysis as described previously [1-2]. The processed samples were visualized using a JEOL JEM 1010 transmission electron microscope (JEOL, Japan) that operated at 80 kV.

SUPPLEMENTARY FIGURES

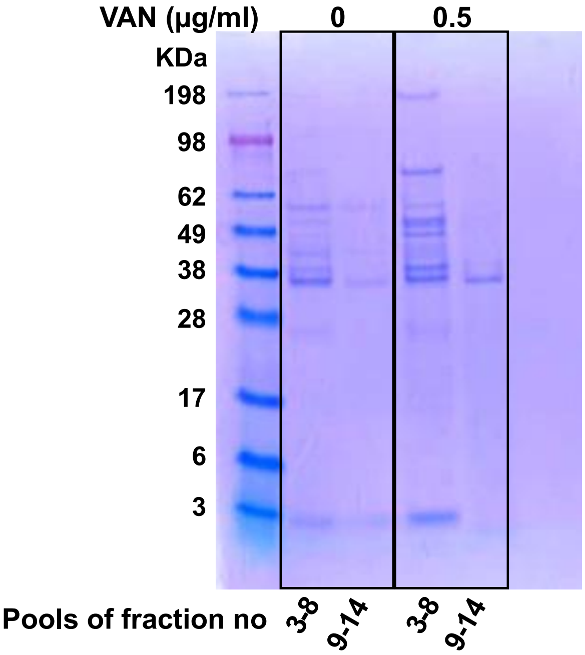

Figure S1. Coomassie blue stained protein gel of pooled MRSA-derived MVs fractions. Upon fractionation of MVs by density gradient ultracentrifugation, fractions (total: 25 fractions) were sequentially collected, and their Coomassie Blue staining pattern on SDS-PAGE were analysed. The fractions showing the same protein profile were pooled and subjected to the proteomic analysis.

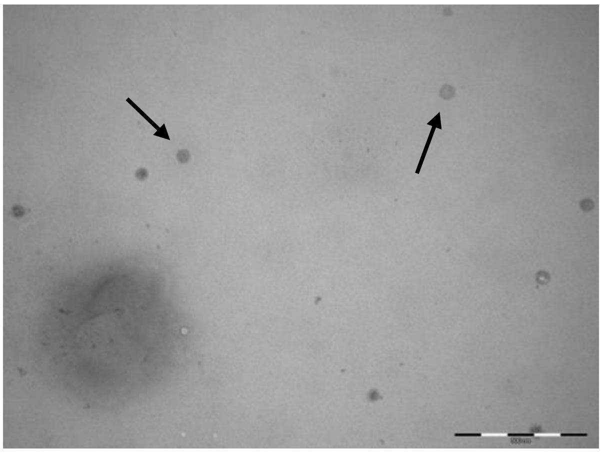

Figure S2. TEM of MVs isolated from MRSA (TCH 1516) after OptiPrep. Representative MVs are marked with arrows. Scale bar is 500 nm.

DATASET LEGEND (See Excel Files)

Dataset 1. List of identified MRSA-derived MV proteins obtained from bacteria grown in BHI in the absence or presence of sub-MIC VAN (0.5 mg/L). The results were obtained from pooled MVs purified from multiple isolations. Protein abundance is presented as the area under the curve (AUC).

Dataset 2. List of identified MRSA-derived MV proteins with increased expression (>2 fold) in the presence of sub-MIC VAN relative to no VAN.

SUPPLEMENTARY REFERENCES

1. Wagner, T., *et al*., *Enterococcus faecium* produces membrane vesicles containing virulence factors and antimicrobial resistance related proteins. Journal of proteomics, 2018. **187**: p. 28 38.
2. Cavanagh, J.P., *et al.*, Comparative exoproteome profiling of an invasive and a commensal *Staphylococcus haemolyticus* isolate. Journal of proteomics, 2018. **197**: 106-114.
